# Effectiveness and costs of phototest in dementia and cognitive impairment screening

**DOI:** 10.1186/1471-2377-11-92

**Published:** 2011-07-29

**Authors:** Cristobal Carnero-Pardo, Beatriz Espejo-Martinez, Samuel Lopez-Alcalde, Maria Espinosa-Garcia, Carmen Saez-Zea, Rosa Vilchez-Carrillo, Elisa Hernandez-Torres, Jose L Navarro-Espigares

**Affiliations:** 1 Cognitive Behavioral Neurology Unit, Service of Neurology, Virgen de las Nieves University Hospital, Carretera de Jaen s/n, 18013 - Granada, Spain; 2 FIDYAN Neurocenter, Mozart s/n, Edf. Zafiro, 18004 - Granada, Spain; 3 Service of Neurology, La Mancha Center Hospital Complex, Alcazar de San Juan, 13600 - Ciudad Real, Spain; 4 Departament of Psychobiology, School of Psychology, University of Jaen, Campus Las Lagunillas s/n, 23071 - Jaen, Spain; 5 Management Control Section, Virgen de las Nieves University Hospital, Carretera de Jaen s/n, 18013 - Granada, Spain

## Abstract

**Background:**

To assess and compare the effectiveness and costs of Phototest, Mini Mental State Examination (MMSE), and Memory Impairment Screen (MIS) to screen for dementia (DEM) and cognitive impairment (CI).

**Methods:**

A phase III study was conducted over one year in consecutive patients with suspicion of CI or DEM at four Primary Care (PC) centers. After undergoing all screening tests at the PC center, participants were extensively evaluated by researchers blinded to screening test results in a Cognitive-Behavioral Neurology Unit (CBNU). The gold standard diagnosis was established by consensus of expert neurologists. Effectiveness was assessed by the proportion of correct diagnoses (diagnostic accuracy [DA]) and by the kappa index of concordance between test results and gold standard diagnoses. Costs were based on public prices and hospital accounts.

**Results:**

The study included 140 subjects (48 with DEM, 37 with CI without DEM, and 55 without CI). The MIS could not be applied to 23 illiterate subjects (16.4%). For DEM, the maximum effectiveness of the MMSE was obtained with different cutoff points as a function of educational level [k = 0.31 (95% Confidence interval [95%CI], 0.19-0.43), DA = 0.60 (95%CI, 0.52-0.68)], and that of the MIS with a cutoff of 3/4 [k = 0.63 (95%CI, 0.48-0.78), DA = 0.83 (95%CI, 0.80-0.92)]. Effectiveness of the Phototest [k = 0.71 (95%CI, 0.59-0.83), DA = 0.87 (95%CI, 0.80-0.92)] was similar to that of the MIS and higher than that of the MMSE. Costs were higher with MMSE (275.9 ± 193.3€ [mean ± sd euros]) than with Phototest (208.2 ± 196.8€) or MIS (201.3 ± 193.4€), whose costs did not significantly differ. For CI, the effectiveness did not significantly differ between MIS [k = 0.59 (95%CI, 0.45-0.74), DA = 0.79 (95%CI, 0.64-0.97)] and Phototest [k = 0.58 (95%CI, 0.45-0.74), DA = 0.78 (95%CI, 0.64-0.95)] and was lowest for the MMSE [k = 0.27 (95%CI, 0.09-0.45), DA = 0.69 (95%CI, 0.56-0.84)]. Costs were higher for MMSE (393.4 ± 121.8€) than for Phototest (287.0 ± 197.4€) or MIS (300.1 ± 165.6€), whose costs did not significantly differ.

**Conclusion:**

MMSE is not an effective instrument in our setting. For both DEM and CI, the Phototest and MIS are more effective and less costly, with no difference between them. However, MIS could not be applied to the appreciable percentage of our population who were illiterate.

## Background

There is currently no evidence to support screening for cognitive impairment (CI) or dementia (DEM) in asymptomatic subjects. However, most clinical practice guidelines recommend maintaining an alert attitude and using screening tests in suspected cases for the early identification of these patients in primary care (PC) [1-3].

Short cognitive tests are considered more appropriate screening instruments for CI and DEM [4]. The *Mini Mental State Examination *(MMSE) [5] is the most widely used but has various drawbacks [4,5], being long and complex and requiring the ability to read and perform tasks with paper and pencil. Other shortcomings include: non-normal distribution of results, ceiling effect, high influence of socio-educational variables, and modest reliability [6] and validity, especially for CI [7]. These limitations have greater impact in populations with a low educational level, for which its use has been contraindicated by many authors [8,9]. The absence of a standardized version is an additional problem in Spain, where there are multiple versions that differ in words to be recalled, sentences to be repeated, drawings to be copied, and the order of items [10]. The lack of consensus on cutoff points led some authors [11] to recommend different cutoff scores as a function of literacy and educational level (17/18 for illiterates, 20/21 for individuals without completed primary schooling; 23/24 for individuals with primary schooling or higher), while the NORMACODEM group [12] proposed applying a single cutoff value of 24/25 points and correcting the score according to age and educational level. A further limitation of the MMSE in our setting is that it has not been studied in Spain for CI. Despite these drawbacks, MMSE remains the most widely accepted reference test and has even been used to regulate treatment with anticholinesterases.

Various instruments have been developed to overcome the limitations of the MMSE, reducing the complexity, application time, and influence of socio-educational variables and improving the performance for CI. Instruments recommended for use in PC [5,13,14] include the General Practitioner Assessment of Cognition (GPCOG) [15], Mini-Cog [16], and Memory Impairment Screen (MIS) [17]. Mini-Cog and GPCOG have not been validated in Spain and are not suitable for use in people with a low educational level because they require the use of paper and pencil. There have been three studies in Spain on the MIS [18-20], which evaluates free recall of 4-word and recall facilitated by semantic cues and includes a distracting task during the 2-minute interval between reading and recall. The MIS requires the ability to read and cannot be applied to illiterates. All three Spanish studies were conducted in hospital populations, except for a wide convenience sample of volunteers that served as controls in one study [20]. There was no consensus on cutoff values, with the first two studies recommending a cutoff score of 4/5 for DEM [19,20] but the third recommending 3/4 for DEM and 4/5 for CI [18].

**Figure 1:**
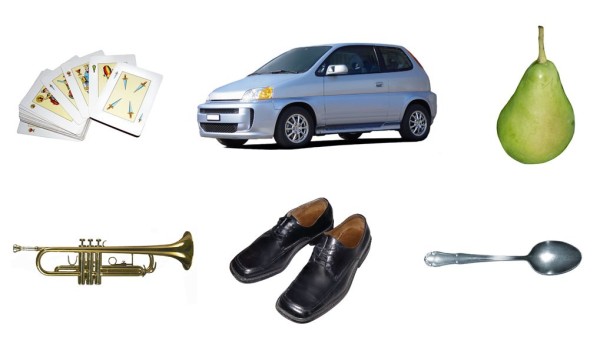
**Phototest laminated sheet (version A)**.

The Phototest (Additional File 1, http://www.fototest.es) [21,22] is a very simple and short instrument (< 3 minutes) that can be applied to illiterates and assesses multiple cognitive fields (language, executive function, episodic memory). It comprises three parts: a naming task with six color photographs of common objects in prototypic position (Figure 1); a verbal fluency test (names of people) demonstrated to be uninfluenced by educational level [23], and, finally, free recall and recall facilitated by cues using the 6 objects in the naming test. There are 2 parallel versions of the test. Version A is usually applied in Spain, but version B (Additional File 2) is more suitable in English-speaking countries because the first two objects in version A are virtually homophonous in English (cards, car). Phototest results are normally distributed and are not influenced by educational level [24]. It has shown good test-retest and interobserver reliability [24], and various studies have reported that cutoff scores of 26/27 and 28/29 points give adequate discriminative validity for DEM and CI, respectively [22,25,26].

The objective of this study was to assess the effectiveness and associated costs of utilizing the Phototest, MMSE, and MIS as screening tests for DEM and CI in PC.

## Methods

### Design

Phase III study of diagnostic test assessment [27] with a paired design (all screening tests were applied to all subjects), with complete verification (all subjects underwent the standard diagnostic procedure) [28].

### Setting

Four PC centers in the Metropolitan District of North Granada Area (Southern Spain).

### Study population

Consecutive patients attended in PC from February 1 2008 to January 31 2009 who aroused suspicion of CI or DEM.

### Inclusion criteria

Inclusion criteria were: suspicion of CI or DEM, based on subjective complaints of memory loss or cognitive alteration, similar complaints made by a relative or informer, or observation by physicians of suspicious signs or symptoms. Exclusion criteria were previous enrolment in this study or previous diagnosis of CI or DEM. There were no age limits, and sensory or motor deficits or other previous conditions were not reasons for exclusion.

### Procedure

All three screening tests were applied in a balanced manner to all participants. Regardless of their test results, all subsequently visited the Cognitive Behavioral Neurology Unit (CBNU) of the Neurology Department of Virgen de las Nieves University Hospital, Granada for behavioral tests (Spanish adaptation of the NPI [29]), functional tests (Barthel index [30], Lawton-Brody scale [31], and Pfeffer's FAQ [32]), and a detailed neuropsychological examination (orientation, executive attention/function [digit span, similarities, verbal fluency], verbal memory [word list], language [abbreviated Boston naming test [33], comprehension of instructions, semantic verbal fluency], visual-spatial functions [drawing, copying], calculation, and motor praxis). All of these tests were conducted by researchers (SLA, MEG) blinded to the PC results. None of the screening tests applied in PC were administered in the CBNU evaluation. The maximum time interval between assessments in the PC center and CBNU was two weeks. Two expert neurologists in cognitive-behavioral neurology (CCP, BME) provided the *gold standard *diagnosis by consensus, based on the CBNU evaluations and a detailed clinical assessment; they classified subjects as: Non-CI (NoCI), CI non-DEM (CInD) (criteria for mild cognitive impairment of the Spanish Neurology Society Neurology and Behavior Study Group [34]), or DEM (DSM-IVR criteria [35]); any lack of consensus was resolved by the decision of a third neurologist (RVC). All three experts were blinded to the PC screening test results.

### Statistical analysis

The effectiveness of the screening tests was assessed by establishing the Sensitivity (Sn) and Specificity (Sp), diagnostic accuracy (DA = proportion of correct diagnoses), and the *kappa *index of diagnostic concordance between the screening test result, considering recommended cutoff points, and the gold standard diagnosis [36]. Effectiveness was calculated for DEM *versus *non-DEM (NoCI + CInD) and for CI (DEM + CInD) *versus *NoCI. MMSE results for DEM diagnosis were independently assessed following the recommendations of Escribano-Aparicio et al [11] and the NORMACODEM study [12], separately analyzing crude scores and scores corrected by age and educational level. Because there are no formal recommendations for CI diagnosis, we applied the 26/27 cutoff point used in a recent Spanish study [37]. MIS results were analyzed using two different cutoff points for DEM, 3/4 [18] and 4/5 [19,20], and a single cutoff score of 4/5 for CI [18]. Phototest results were analyzed using a cutoff value of 26/27 for DEM and 28/29 for CI [22].

The cost analysis was based on the perspective of our healthcare organization and took no account of the direct costs of patients/carers, indirect or intangible costs, or of the effects of diagnostic delay or false negatives (FNs). For each test, we considered the minimum costs required to reach the correct diagnosis for each condition. Hence, true negatives (TNs) only incurred the cost of the PC consultation, whereas true positives (TPs) and false positives (FPs) also incurred the costs of the CBNU study (professionals [neurologist, neuropsychologist, nurse] and of the minimum complementary tests recommended by the Spanish Neurology Society [analytical, cranial CT-scan]), and FNs required at least two PC consultations plus the CBNU evaluation. The cost of the PC consultation was taken from published rates and prices of the Andalusian public healthcare system (Order of October 14 2005). The costs of examinations in the CBNU were based on the hospital's financial accounts (Table 1). Calculations were performed for each instrument on the minimum cost for the whole sample, cost per correct diagnosis, and average cost per subject.

**Table 1:** Minimum costs of diagnostic study

PC study		43.5€
Cost of PC physician consultation	43.5€	
**CBNU study**		**390.8€**
Professionals (neurologist+neuropsychologist+nurse)	197.4€	
Additional test (analysis+cranial CT scan)	193.4€	

**Costs per diagnoses**		
True Negative (PC study)		**43.5€**
True Positive (PC study + CBNU study)		**434.4€**
False Positive (PC study + CBNU study)		**434.4€**
False Negative ([PC study × 2] + CBNU study)		**477.9€**

PC: Primary Care; CBNU: Cognitive-Behavior Neurology Unit. CT: Computed Tomography

SPSS version 15.0 (SPSS Inc., Chicago, IL) was used for the data analyses, comparing qualitative variables with the chi-square test or comparison of proportions and quantitative variables with an ANOVA, applying the Bonferroni test in *post-hoc *analysis. Effectiveness was compared among instruments by using the McNemar test for related samples, and costs were compared with a t-test for related samples. P < 0.05 was considered significant, and 95% confidence intervals were calculated for all study variables.

### Formal aspects

The study was approved by the Ethics and Research Committee of the Virgen de las Nieves University Hospital, and written informed consent was obtained from all participants. The study design and report writing complied with the STARD recommendations for diagnostic test studies [38] and the recommendations of the *Food and Drug Administration *for reporting diagnostic study results [36].

## Results

### Sample characteristics

**Figure 2:**
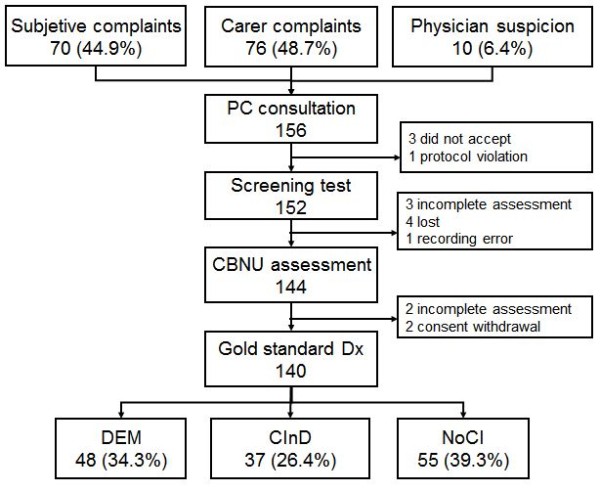
**Flow diagram of study participants**. PC: Primary Care; CBNU: Cognitive Behavioral Neurology Unit; DEM: Dementia; CInD: Cognitive impairment without dementia; NoCI: No cognitive impairment.

The four PC centers in the study serve a population of 66,713 people, of whom around 16.7% are ≥65 years old [39]. During the study period, PC physicians reported suspicion of CI or DEM in 156 patients, due to subjective complaints in 70 cases (44.9%), third-party complaints in 76 (48.7%), and initial detection by the physicians themselves in 10 (6.4%) cases. Out of the 156 patients enrolled in the study, 16 were excluded: 3 did not give consent and 2 withdrew during the study; 4 were lost to the follow-up; and 5 were not fully evaluated, due to sequelae of stroke or traumatic brain injury and/or sensory impairment (amaurosis, anacusis), a protocol violation, and a recording error. Out of the 140 patients completing the study, 48 (34.3%) had DEM, 37 (26.4%) CInD, while 55 (39.3%) had neither CI nor DEM (NoCI) (Figure 2).

Table 2 shows the socio-demographic characteristics and screening test results of the participants, stratified by cognitive diagnosis. Stratifications by "age" and "years of education" were done for score corrections according to NORMACODEM recommendations [12], and "educational level" categories were those used by Escribano-Aparicio et al [11] to define different recommended cutoff points.

**Table 2:** Socio-demographic characteristics and screening test results by diagnostic group

	Total	CI	NoCI	CInD	DEM	NoDEM
**N° Subjects**	140	85	55	37	48	92
**N° females**	101 (72.1)	60 (70.6)	41 (74.5)	23 (62.2)	37 (77.1)	64 (69.6)
Age in yrs*	72.5 ± 11.3	77.3 ± 7.6	64.9 ± 12.1	74.1 ± 7.3	79.6 ± 7.0	68.6 ± 11.3
≤ 50 yrs	7 (5.0)	0 (0)	7 (12.7%)	0 (0)	0 (0)	7 (7.6)
51-75 yrs	70 (50.0)	33 (37.5)	37 (67.3)	19 (51.4)	14 (27.5)	56 (60.9)
≥ 76 yrs	63 (45.0)	52 (61.2)	11 (20.0)	18 (48.6)	34 (70.8)	29 (31.2)
**Educational Level^#^**						
Illiterates	20 (14.3)	19 (22.4)	1 (1.8)	3 (8.1)	16 (33.3)	4 (4.3)
Incomplete primary	52 (37.1)	35 (41.2)	17 (30.9)	16 (43.2)	19 (39.6)	33 (35.9)
Primary or more	68 (48.6)	31 (36.5)	37 (67.3)	18 (48.6)	13 (27.1)	55 (59.8)
**Years of Education^#^**						
≤8 yrs	72 (51.4)	54 (63.5)	18 (32.7)	19 (51.4)	35 (72.9)	37 (40.2)
9-17 yrs	61 (43.6)	31 (36.5)	30 (54.5)	18 (48.6)	13 (21.3)	48 (52.2)
≥ 18 yrs	7 (5.0)	0 (0)	7 (12.7)	0 (0)	0 (0)	7 (7.6)
**MMSE^#^**	19.9 ± 5.7	17.2 ± 5.3	24.1 ± 3.1	21.8 ± 3.5	13.8 ± 3.6	23.2 ± 3.4
**MMSEc^@^**	20.8 ± 5.2	18.5 ± 5.1	24.4 ± 2.9	22.8 ± 3.2	15.2 ± 3.7	23.7 ± 3.1
**MIS^#^**	4.3 ± 2.9 [117]	2.6 ± 2.6 [64]	6.2 ± 1.8 [53]	4.1 ± 2.5 [34]	1.0 ± 1.5 [30]	5.4 ± 2.3 [87]
**Phototest^#^**	29.0 ± 7.6	25.4 ± 6.8	34.6 ± 5.1	30.5 ± 4.8	21.5 ± 5.5	32.9 ± 5.4

CI: cognitive impairment (CInD and DEM). NoCI: no cognitive impairment. CInD: cognitive impairment without dementia. DEM: dementia. NoDEM: no dementia (NoCI and CInD).

MMSE: MiniMental State Examination. MMSEc: MMSE with corrected scores. MIS: Memory Impairment Screen.

The figures are n° of subjects (percentage) or mean ± sd; figures in square brackets = n° subjects able to take the MIS

* DEM > CInD > NoCI; DEM > NoDEM; CI > NoCI (p < 0.0001 all comparisons)

# NoCI > CInD > DEM; NoDEM > DEM; NoCI > CI (p < 0.0001 all comparisons)

@ (NoCI = CInD) > DEM; NoDEM > DEM; NoCI > CI; (NoCI vs CInD: ns; remaining comparisons, p < 0.0001)

The age of the study sample was 72.5 ± 11.3 yrs (mean ± sd), and 72.1% were female; 20 (14.3%) were illiterate and 52 (37.1%) had not completed primary schooling. There was no significant gender difference among diagnostic groups, but subjects with DEM were older and had a lower educational level than those with CInD or NoCI (p < 0.0001 for all comparisons). The groups significantly differed in test results in the order NoCI > CInD > DEM (p < 0.0001 for all comparisons) with the exception of the corrected MMSE score, in which the difference between NoCI and CInD groups did not reach significance (p = 0.08).

### Effectiveness and cost for DEM

For DEM (Table 3), the diagnostic concordance and accuracy of MMSE were low (k = 0.30 [95% Confidence Interval (95%CI), 0.16 to 0.44]; DA = 0.59 [95%CI, 0.46 to 0.73]) when NORMACODEM study criteria were used [12]) and were not improved when scores were corrected by age and educational level (k = 0.29 [95%CI, 0.15 to 0.43]; DA = 0.58 [95%CI, 0.46 to 0.72]). The diagnostic concordance and accuracy were improved but remained moderate (k = 0.50 [95%CI, 0.36 to 0.64]; DA = 0.74 [95%CI, 0.60 to 0.89]) when the recommendations of Escribano-Aparicio et al [11] for populations with low educational level were followed.

**Table 3:** Effectiveness and cost of the screening tests for dementia

Test	CoP	Sn	Sp	DA	k	**Total Cost **(€)	Cost/CD (€)	**Mean cost **(€)
**MMSE**	**24/25**	1.00 (0.93-1.00)	0.38 (0.28-0.49)	0.59 (0.46-0.73)	0.30 (0.16-0.44)	47134.5	585.2	336.7 ± 169.9
**MMSEc**	**24/25**	1.00 (0.93-1.00)	0.37 (0.27-0.48)	0.58 (0.46-0.72)	0.29 (0.15-0.43)	47525.4	570.6	339.5 ± 168.2
**MMSE**	*	0.96 (0.86-0.99)	0.62 (0.51-0.72)	0.74 (0.60-0.89)	0.50 (0.36-0.64)	38621.7	377.9	275.9 ± 193.3
**MIS#**	**3/4**	0.93 (0.78-0.99)	0.80 (0.71-0.88)	0.83 (0.67-1.00)	0.63 (0.48-0.78)	23548.6	242.5	201.3 ± 193.4
**MIS#**	**4/5**	0.96 (0.78-1.00)	0.71 (0.61-0.80)	0.78 (0.73-1.00)	0.54 (0.38-0.69)	26632.5	295.6	227.6 ± 196.4
**Phototest**	**26/27**	0.81 (0.67-0.91)	0.89 (0.81-0.95)	0.86 (0.72-1.00)	0.70 (0.57-0.83)	29153.7	242.1	208.2 ± 196.8

CoP: Cutoff point; Sn: sensitivity; Sp: specificity; DA: diagnostic accuracy (proportion of correct diagnoses); k: kappa index. Cost/CD: total cost divided by n° correct diagnoses. Mean cost: mean ± sd. In brackets, 95% confidence interval.

MMSE: MiniMental State Examination. MMSEc: MMSE with corrected scores. MIS: Memory Impairment Screen

*Cutoff points: 17/18: illiterates; 20/21: literate but did not complete primary schooling; 23/24: primary schooling or higher

#Results for the 117 subjects who completed the MIS

MIS could not be applied to 23 subjects (16.4% of the sample), due to illiteracy (20) or minimum reading capacity associated with visual deficit (3). Among the 117 tested with MIS, a good diagnostic concordance (k = 0.63 [95%CI, 0.48 to 0.78]; DA = 0.83 [95%CI, 0.67 to 1.0]) was obtained with a DEM cutoff score of 3/4, non-significantly better than the concordance obtained with a cutoff of 4/5 (k = 0.54 [95%CI, 0.38 to 0.69]; DA = 0.78 [95%CI, 0.73 to 1.0]); the result was superior with either cutoff score to any of the results obtained with MMSE. The diagnostic concordance of Phototest was substantial (k = 0.70 [95%CI, 0.57 to 0.83]; DA = 0.86 [95%CI, 0.72 to 1.00]), did not significantly differ from that of MIS, and was significantly superior to that of MMSE.

Sn and Sp values of Phototest (Sn = 0.81 [95%CI, 0.67 to 0.91], Sp = 0.89 [95%CI, 0.81 to 0.95]) did not significantly differ from those of MIS with the cutoff point of 3/4 (Sn = 0.93 [95%CI, 0.78 to 0.99], χ^2 ^= 1.78, ns; Sp = 0.80 [95%CI, 0.71 to 0.88, χ^2 ^= 2.11, ns]) but differed from those of MMSE (Sn = 0.96 [95%CI, 0.86 to 0.99], χ^2 ^= 4.0, p < 0.05; Sp = 0.62 [95%CI, 0.51 to 0.72], χ^2 ^= 15.6, p < 0.001) (Table 4).

**Table 4:** Comparison of the effectiveness of screening tests for dementia, considering the most favorable MMSE and MIS approaches

	DEM			NoDEM		
	
Test	Phototest ≤26	Phototest ≥27	Total	Phototest ≤26	Phototest ≥27	Total
MMSE* +	**38**	8	46	4	31	35

MMSE* -	**1**	1	2	6	51	57

**Total**	**39**	9	48	10	82	92

	χ**^2 ^**= 4; p < 0.05			χ**^2 ^**= 15.6, p < 0.001		

**MIS ≤3# **	**21**	7	28	5	12	17

**MIS ≥4#**	**2**	0	2	5	65	70

**Total#**	**23**	7	30	10	77	87

	χ**^2 ^**= 1.78; n.s.			χ**^2 ^**= 2.11; n.s.		

DEM: dementia; NoDEM: No dementia. MMSE: MiniMental State Examination. MIS: Memory Impairment Screen

Values are the n° of subjects

*Cutoff points: 17/18: illiterates; 20/21: literate but did not complete primary schooling; 23/24: primary schooling or higher

#Results for the 117 subjects who completed the MIS

**Figure 3:**
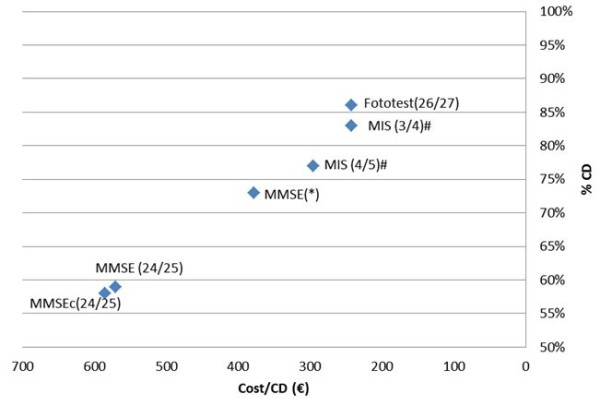
**Cost per correct diagnosis for dementia**. MMSE: MiniMental State Examination. MMSEc: MMSE with corrected scores. MIS: Memory Impairment Screen. CD: correct diagnoses. *Cutoff points: 17/18: illiterates; 20/21: literate but did not complete primary schooling; 23/24: primary schooling or higher #Results for the 117 subjects who completed the MIS

The mean cost of applying Phototest (208.2 ± 196.8 €) did not significantly differ from that of the optimal MIS approach (201.3 ± 193.4 €, t = 1.58, ns) but was significantly lower versus the optimal MMSE approach (275.9 ± 193.3 €, t = 4.2, p < 0.05), as depicted in Figure 3.

### Effectiveness and cost in CI

For all instruments, the predictive validity was lower for CI than for DEM (Table 5). No difference was found between results obtained with MIS (k = 0.59 [95%CI, 0.45 to 0.74]; DA = 0.79 [95%CI, 0.64 to 0.97]) and Phototest (k = 0.58 [95%CI, 0.45 to 0.74]; DA = 0.78 [95%CI, 0.64 to 0.95]), and both results were significantly higher *versus *MMSE, using both crude scores (k = 0.24 [95%CI, 0.06 to 0.43]; DA = 0.69 [95%CI, 0.55 to 0.84]) and scores corrected by age and educational level (k = 0.27 [95%CI, 0.09 to 0.45]; DA = 0.69 [95%CI, 0.56 to 0.84]), which did not significantly differ between them.

**Table 5:** Effectiveness and cost of screening tests for cognitive impairment

Test	CoP	Sn	Sp	DA	k	Total Cost (€)	Cost/CD	Mean cost (€)
**MMSE**	**26/27**	0.96 (0.90-0.99)	0.24 (0.13-0.37)	0.69 (0.55-0.84)	0.24 (0.06-0.43)	55821.3	577.9	398.7 ± 114.2
**MMSEc**	**26/27**	0.96 (0.90-0.99)	0.27 (0.16-0.41)	0.69 (0.56-0.84)	0.27 (0.09-0.45)	55582.8	575.4	393.4 ± 121.8
**MIS#**	**4/5**	0.73 (0.61-0.84)	0.87 (0.75-0.94)	0.79 (0.64-0.97)	0.59 (0.45-0.74)	33582.9	363.3	287.0 ± 197.4
**Phototest**	**28/29**	0.69 (0.58-0.79)	0.93 (0.82-0.98)	0.78 (0.64-0.95)	0.58 (0.45-0.74)	42014.0	379.8	300.1 ± 195.6

CoP: Cutoff point; Sn: sensitivity; Sp: specificity; DA: diagnostic accuracy (proportion correct diagnosis); k: kappa index. Cost/CD: total cost divided by n° correct diagnoses. Mean cost: mean ± sd. In brackets, 95% confidence interval.

MMSE: MiniMental State Examination. MMSEc: MMSE with corrected scores. MIS: Memory Impairment Screen

#Results for the 117 subjects who completed the MIS

Sn and Sp values did not significantly differ between MIS (S = 0.73 [95%CI, 0.61 to 0.94]; Sp = 0.87 [95%CI, 0.75 to 0.94]) and Phototest (S = 0.69 [95%CI, 0.58 to 0.79], χ^2 ^= 1.23, ns; Sp = 0.93 [95%CI, 0.82 to 0.98], χ^2 ^= 0.31, ns) and were significantly lower for MMSE (Table 6).

**Table 6:** Comparison of the effectiveness of screening tests for cognitive impairment (most favorable alternatives for MMSE and MIS)

	CI			NoCI		
	
Test	Phototest≤28	Phototest≥29	Total	Phototest≤28	Phototest≥29	Total
**MMSEc ≤26**	58	24	82	4	36	40

**MMSEc ≥27 **	1	2	3	0	15	15

**Total**	59	26	85	4	51	55

	χ^2 ^= 19.36; p < 0.001			χ^2 ^= 32.11; p < 0.001		

**MIS ≤4# **	38	9	47	0	7	7

**MIS ≥5# **	4	13	17	4	42	46

**Total#**	42	22	64	4	49	53

	χ^2 ^= 1.23; ns			χ^2 ^= 0.31; ns		

CI: cognitive impairment; NoCI: No cognitive impairment. MMSE: MiniMental State Examination. MMSEc: MMSE with corrected scores. MIS: Memory Impairment Screen.

Values are the n° of subjects

#Results referred to the 117 subjects who completed the MIS

**Figure 4:**
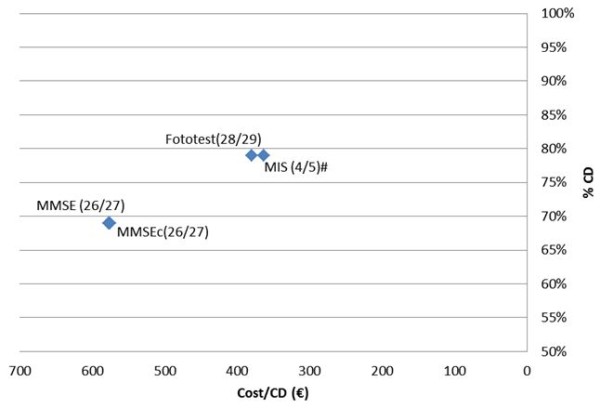
**Cost per correct diagnosis for cognitive impairment**. MMSE: MiniMental State Examination. MMSEc: MMSE with corrected scores. MIS: Memory Impairment Screen. CD: correct diagnosis. *Cutoff points: 17/18: illiterates; 20/21: literate but did not complete primary schooling; 23/24: primary schooling or higher. #Results for the 117 subjects who completed the MIS

The mean cost of MMSE (398.7 ± 114.2 €) was significantly higher than the cost of MIS (287.0 ± 197.4 €; t = 6.01, p < 0.0001) and Phototest (300.1 ± 195.6 €; t = 6.26, p < 0.0001), which did not significantly differ between them (t = 0.47, ns), as depicted in Figure 4.

## Discussion

This study of CI and DEM screening test assessment in an urban population with a low educational level shows that MMSE is not very useful for DEM screening, despite its widespread utilization. Its results were improved by adopting the correction criteria proposed by Escribano-Aparicio et al for populations with low educational level, but they remained significantly inferior to those obtained with Phototest and MIS.

Phototest, a short instrument that is rapid and easy to use, showed a substantial diagnostic concordance for DEM, correctly classifying 86% of the study sample, similar to the percentage obtained using MIS with a cutoff score of 3/4. However, unlike Phototest, MIS requires reading ability and could not be applied to an appreciable proportion of our study population (16.4%).

Although all instruments were significantly less useful for CI than for DEM, Phototest correctly classified 78% of patients as CI or non-CI, a similar percentage to that obtained with MIS. MMSE was the least useful instrument to screen for CI.

To our knowledge, this is the first study of CI and DEM screening tests to evaluate the costs associated with utilization of the test instruments. MMSE was associated with significantly higher costs for both DEM and CI, with no significant difference in the costs of Phototest and MIS. Considering the whole study sample, there was a difference between Phototest and the optimal MMSE approach of 13,807.3 € for CI screening and 9,468 € for DEM. Extrapolation of this difference to the whole population served by the unit (i.e., around 500,000 *versus *66,713 subjects) indicates that the use of Phototest instead of MMSE would yield savings of around 70,000-135,000 € per year at present prices. Although the present study is not strictly a cost-effectiveness analysis [40], it can be concluded from our data on effectiveness and costs that Phototest is the best option for CI and DEM screening, offering higher effectiveness at lower cost in comparison to MMSE and allowing the assessments of all subjects, unlike MIS. This is valuable information for the efficient allocation of resources when these are limited [41].

The fact that MIS cannot be used with illiterates may appear irrelevant given the tendency to an improvement in educational level and the aim of universal literacy [42]. However, illiteracy remains a worldwide problem, with over 750 million illiterates in 2010 [43], and it is not limited to underdeveloped countries, affecting 3% of adults in the USA. i.e., around 7 million people [44]. Account must also be taken of the increasing frequency of "relative illiteracy" derived from emigration and tourism [45], resulting in large numbers of people who are literate in their mother tongue and can express themselves verbally in the language of their country of residence but have inadequate reading skills in the new language for assessment with instruments that require this ability. This situation is considered to affect 2% of adults in the USA, i.e., around four million people [44]. In short, instruments that do not require reading skills remain necessary to assess the whole population.

The main strengths of this study are the prospective, consecutive, and systematic nature of the recruitment, the long study period, the virtual absence of exclusion criteria, and the low loss index. As a result, the study sample is a faithful reflection of the diagnostic challenges faced under routine clinical conditions in this field of care, making the study naturalistic and pragmatic and allowing robust results that can be considered effectiveness rather than efficacy estimators. Furthermore, the main biases in diagnostic test assessment studies [46] were avoided by the fact that all subjects underwent all screening tests, all received a gold standard diagnosis, regardless of their screening test results, and the screening test results and gold standard diagnoses were all evaluated in a blinded and independent manner.

One study limitation is that it may not be possible to extrapolate the results, especially those for the MMSE, to more educated populations. The low specificity of MMSE was undoubtedly due to the low educational level of our sample. This shortcoming is avoided by the Phototest, whose results are not affected by educational level [24]. The present results for the overall usefulness of the Phototest are virtually identical to those obtained in a previous multicenter study (FOTOTRANS study) conducted under routine clinical conditions, which used the same cutoff points and found a sensitivity of 0.88 and specificity of 0.87 for DEM, and sensitivity of 0.68 and specificity of 0.89 for CI [25,26]. In the FOTOTRANS study, the diagnostic accuracy of the Phototest was similar to that of the Eurotest [47], another valid and reliable cognitive test applicable to illiterates and developed by our group [48,49]. However, the Eurotest requires 7 minutes to complete and is therefore less useful in PC, in which time constraints are crucial [50].

The present results found for MIS with a cutoff point of 3/4 are also very similar to the findings by Perez-Martinez et al in a prospective clinical sample with low educational level (Sn = 0.93, Sp = 0.73) [18] but differ from those reported in two other studies (Sn = 0.74, Sp = 0.96, and Sn = 0.84, Sp = 0.94, respectively) [19,20]. In these two studies, the specificity was higher than the sensitivity, possibly because of the different cutoff point used (4/5), the cross-sectional design of the studies, and the higher overall educational level of the samples, which did not include subjects with CI, facilitating the discrimination between DEM and non-DEM and therefore overestimating the diagnostic usefulness.

Although our sample size may appear to be a limitation, it allowed the precise estimation of proportions (6-8%). Furthermore, the use of a one-year recruitment period allowed seasonal biases to be avoided and ensured greater sample representativeness.

Finally, our economic analysis was simplistic and basic and cannot strictly be considered a cost-effectiveness study [40]. Nevertheless, our approach to minimum costing was real and pragmatic and allowed differential costs to be established among the different screening test options. Our data should facilitate decision-making to improve the assignment of resources. In this regard, our results suggest that MMSE is not suitable for use in our setting and that MIS and Phototest are preferable due to their lower cost and higher effectiveness. The Phototest can be especially recommended because, unlike the MIS, it can be completed by all subjects, including the illiterate.

## Conclusions

In this prospective phase III study of diagnostic test assessment in a population with low educational level, we assessed, in a blinded and independent manner, the effectiveness and cost of CI and DEM screening using three tests: MMSE and MIS, the most widely used tests in PC, and Phototest, a short, easily applied test that can be used in illiterates and whose results are not influenced by educational level. MMSE proved to be the least effective and most expensive option, with a low diagnostic usefulness. The effectiveness of MIS and Phototest was substantially higher than that of MMSE, and their associated costs were lower. However, MIS could not be applied to illiterates, whereas Phototest could be used with the whole sample. The application in PC of Phototest rather than MMSE to screen for CI and DEM in our setting would improve the effectiveness of the screening, reduce the workload of healthcare professionals, and lower the associated costs, permitting a better distribution of resources.

## Competing interests

C. Carnero-Pardo is the creator of the Phototest and Eurotest.

## Authors' contributions

CCP formulated the study design, led the data collection, carried out the analyses, interpreted the results, and wrote the first draft of the manuscript. EHT and JLNE discussed the study design, calculated costs, assisted with the data analyses and results interpretation. BEM, SLA, MEG, CSZ, and RVC discussed the study design, collected data and commented on results. All authors read and approved the final manuscript.

## Pre-publication history

The pre-publication history for this paper can be accessed here:

http://www.biomedcentral.com/1471-2377/11/92/prepub

## Supplementary Material

Additional file 1 **Phototest**. Form with instructions for the application and correction of the Phototest. Click here for file

Additional file 2 **Phototest laminated sheet (version B)**. Laminated sheet with stimulus images in the version for English-speaking countries. Click here for file
