# Supplementary material for: Effectiveness and costs of phototest in dementia and cognitive impairment screening
Source: BMC Neurol. 2011 Jul 29;11:92. doi: 10.1186/1471-2377-11-92 (PMC3160880; doi:10.1186/1471-2377-11-92)
Supplement: Additional file 1 — Phototest. Form with instructions for the application and correction of the Phototest. [file 1471-2377-11-92-S1.PDF]

PHOTOTEST

Name: Age: Date:

1.- Naming (Nam)

Show him/her the sheet of photos and ask him/her to name them;award 1 point for each correct answer; in case of error or no response, indicate the correct name and award no points

(Once this task is finished, withdraw the lamina and keep out of sight of the subject)

2.- Verbal Fluency (FM/FW)

a.- *"I want you to tell me all the names of men/women* that you can remember* (*if the subject is male, say "women", if female, say "men")
(Do not give examples, allow 30 seconds and start recording the time from when he/she says the first name. Give 1 point for each correct name, do not score similar names twice (e.g., Cathy-Catherine; Joe-Joseph; Jenny-Jennifer, etc.)

b- *"I want you to tell me all the names of men/women** that you can remember* (** if the subject is male, say "men", if female, say "women")
(Do not give examples, allow 30 seconds and start recording the time from when he/she says the first name. Give 1 point for each correct name, do not score similar names twice (e.g., Cathy-Catherine; Joe-Joseph; Jenny-Jennifer, etc.)

3.- Recall

a.- Free Recall (FR): "*Do you remember what photos there were in the sheet I showed you before?*". (Maximum 20 seconds); give 2 points for each correct response.

b.- Cued Recall (CR): Offer as a cue and assistance the "category" of images that he/she did NOT remembered spontaneously before, saying for example: "*There was also a fruit, do you remember it?*" . Give 1 point for each correct response.

Category	Photos		Nam	FR*	CR	Fluency Men (FM)	Fluency Women (FW)
	Version A	Version B					
Game	Cards	Domino					
Vehicle	Car	Truck					
Fruit	Pear	Grapes					
Musical Instrument	Trumpet	Fork					
Article of clothing	Shoes	Gloves					
Cutlery	Spoon	Drum					
		Sub-Totals					
Total Fototest (Nam+FR+CR+FM+FW)							

* 2 points for each correct response

Modification of: Carnero Pardo C, et al. Utilidad diagnóstica del Test de las Fotos (Fototest) en deterioro cognitivo y demencia". Neurología 2007; 22 (10): 860-869

English version: Richard Davies

OBSERVATIONS.-
