# Supplementary figures and images for: Effectiveness and costs of phototest in dementia and cognitive impairment screening
Source: BMC Neurol. 2011 Jul 29;11:92. doi: 10.1186/1471-2377-11-92 (PMC3160880; doi:10.1186/1471-2377-11-92)

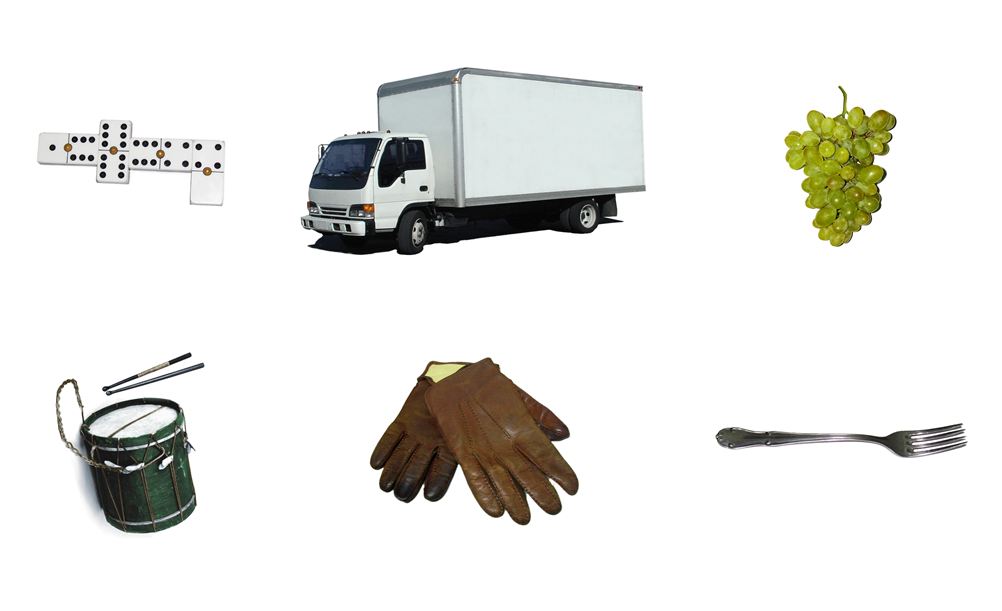

Supplement: Additional file 2 — Phototest laminated sheet (version B). Laminated sheet with stimulus images in the version for English-speaking countries. [file 1471-2377-11-92-S2.JPEG]
